# Migration of Influenza Virus Nucleoprotein into the Nucleolus Is Essential for Ribonucleoprotein Complex Formation

**DOI:** 10.1128/mbio.03315-21

**Published:** 2022-01-04

**Authors:** Sho Miyamoto, Masahiro Nakano, Takeshi Morikawa, Ai Hirabayashi, Ryoma Tamura, Yoko Fujita-Fujiharu, Nanami Hirose, Yukiko Muramoto, Takeshi Noda

**Affiliations:** a Laboratory of Ultrastructural Virology, Institute for Frontier Life and Medical Sciences, Kyoto University, Kyoto, Japan; b Laboratory of Ultrastructural Virology, Graduate School of Biostudies, Kyoto University, Kyoto, Japan; c CREST, Japan Science and Technology Agency, Kawaguchi, Saitama, Japan; Boston University School of Medicine

**Keywords:** RNP, assembly, influenza virus, nucleolus

## Abstract

Influenza A virus double-helical ribonucleoprotein complex (RNP) performs transcription and replication of viral genomic RNA (vRNA). Although RNP formation occurs in the nuclei of virus-infected cells, the nuclear domains involved in this process remain unclear. Here, we show that the nucleolus is an essential site for functional RNP formation. Viral nucleoprotein (NP), a major RNP component, temporarily localized to the nucleoli of virus-infected cells. Mutations in a nucleolar localization signal (NoLS) on NP abolished double-helical RNP formation, resulting in a loss of viral RNA synthesis ability, whereas ectopic fusion of the NoLS enabled the NP mutant to form functional double-helical RNPs. Furthermore, nucleolar disruption of virus-infected cells inhibited NP assembly into double-helical RNPs, resulting in decreased viral RNA synthesis. Collectively, our findings demonstrate that NP migration into the nucleolus is a critical step for functional RNP formation, showing the importance of the nucleolus in the influenza virus life cycle.

## INTRODUCTION

Influenza A virus, belonging to the *Orthomyxoviridae* family, possesses eight-segmented, single-stranded, negative-sense RNA as its genome. Each viral genomic RNA (vRNA) segment exists as a ribonucleoprotein complex (RNP) associated with multiple nucleoproteins (NPs) and a heterotrimeric RNA-dependent RNA polymerase complex composed of PB2, PB1, and PA subunits (1). The RNPs, which are flexible double-stranded helices (width, ∼10 nm; length, 30 to 120 nm) (2), are responsible for transcription and replication of the vRNAs. Upon transcription, vRNA is transcribed into 5′-capped and 3′-polyadenylated mRNA by the polymerase complex in a primer-dependent manner. During genome replication, the vRNA is copied into a cRNA replicative intermediate by a *cis*-acting viral polymerase complex, and the cRNA acts as a template for generating more vRNAs, with involvement of a *trans*-activating/*trans*-acting viral polymerase complex (3, 4). These replication processes are concomitant with RNP assembly; the 5′ terminals of the nascent vRNA and cRNA are associated with a newly synthesized viral polymerase complex that is sequentially coated with multiple NPs and assembled into double-helical vRNPs and cRNPs, respectively (5).

Unlike most RNA viruses, influenza A virus transcribes and replicates its genome in the nuclei of virus-infected cells (6). Accordingly, influenza A virus transcription, replication, and RNP formation heavily rely on host nuclear machineries. Upon initiation of vRNA transcription, the viral polymerase complex in the RNP binds to the carboxy-terminal domain of host RNA polymerase II (Pol II) (7). Then, the PB2 subunit binds to the 5′-cap structure of host pre-mRNAs or small nuclear/nucleolar RNAs (8, 9), and the PA subunit cleaves and snatches the 5′-capped fragment for use as a primer (10–12). The requirement of Pol II for initiation of viral mRNA synthesis indicates that the genome transcription takes place in the nucleoplasm, near host Pol II localization. Genome replication and double-helical RNP formation reportedly involves several intranuclear host factors, such as minichromosome maintenance helicase complex, UAP56, Tat-SF1, and ANP32 (13). In addition, recent studies have demonstrated the importance of the intranuclear proteins fragile X mental retardation protein (FMRP), protein kinase C, and LYAR in the replication-coupled RNP assembly (14–16). However, since these host proteins are localized in different intranuclear domains, the subnuclear site of vRNA replication and RNP formation remains unidentified.

Previously, we showed that a mutant influenza A virus lacking the hemagglutinin (HA) vRNA segment efficiently incorporates 18S and 28S ribosomal RNAs (rRNAs) into progeny virions instead of the omitted HA vRNA and that those rRNAs are associated with viral NPs and form RNP-like structures (17). Considering that NPs are localized to not only the nucleus but also the nucleolus (18, 19), we hypothesized that assembly of NPs into a double-helical RNP relies on the nucleolus, the site of rRNA transcription, pre-rRNA processing, and ribosomal assembly. Thus, we employed several microscopic and biochemical approaches to investigate the importance of the nucleolus in functional RNP formation using NP mutants lacking an intrinsic nucleolar localization signal, as well as a compound that causes nucleolar disruption in cells.

## RESULTS

### Nucleolar localization of NP in virus-infected cells.

**FIG 1:**
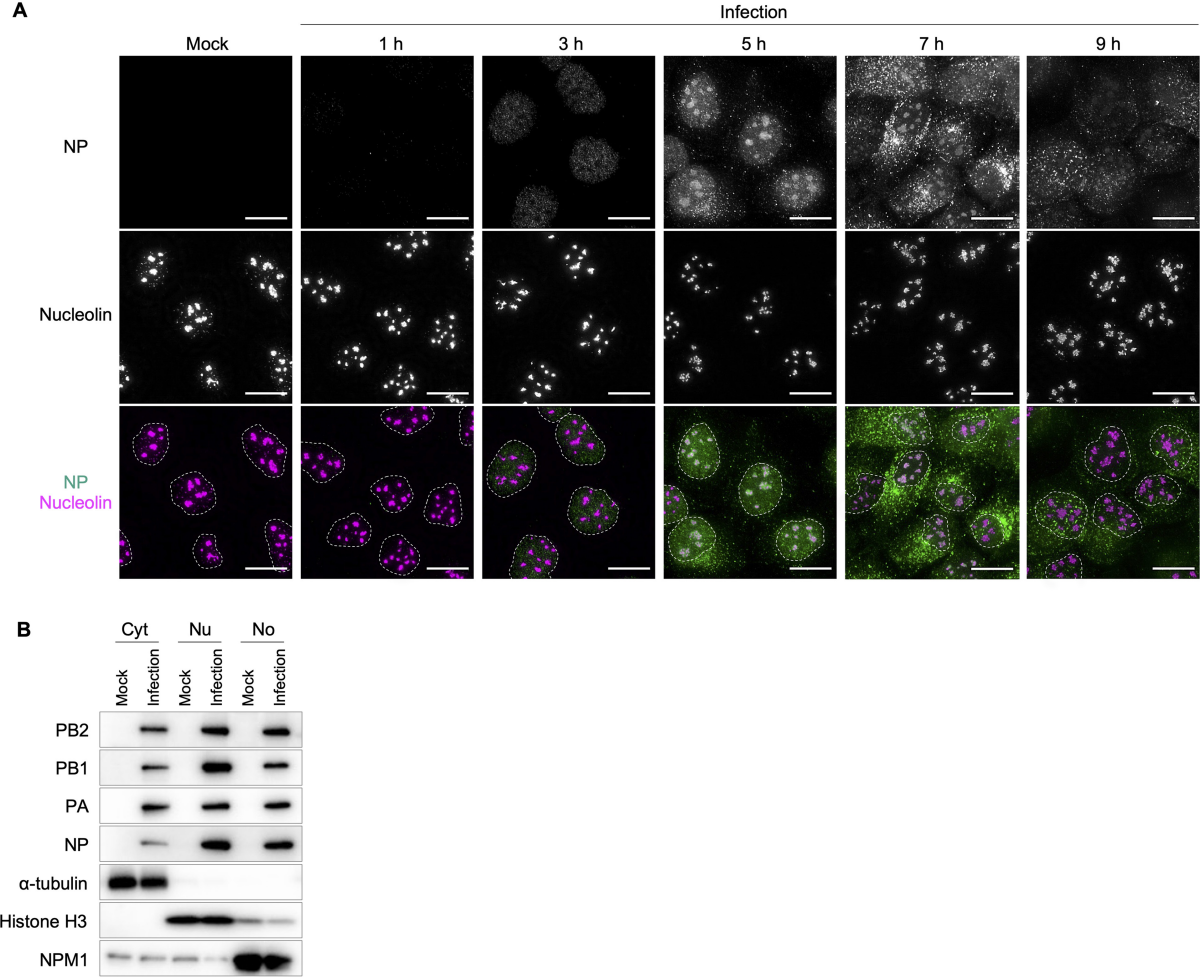
Nucleolar localization of RNP components in influenza virus-infected cells. (A) Subcellular translocation of NPs in mock-infected or influenza virus-infected (MOI = 5) cells. NP and nucleolin were immunostained after protease treatment of fixed and permeabilized cells. Nuclei are marked by dashed circles. Scale bars, 20 μm. (B) Subcellular fractionation of the infected cells. Mock-infected or influenza virus-infected MDCK cells at an MOI of 5 were fractionated into cytoplasmic (Cyt), nucleoplasmic (Nu), and nucleolus (No) fractions at 4 hpi. Approximately 5 μg total protein was analyzed by Western blotting of viral proteins and cell fraction-specific markers α-tubulin (Cyt), histone H3 (Nu), and NPM1 (No). All images are representative of three independent experiments.

To confirm that *de novo*-synthesized NP is localized to the nucleoli of virus-infected cells, Madin-Darby canine kidney (MDCK) cells were infected with influenza A virus and fixed over time. The virus-infected cells were treated with a protease to remove highly condensed host nucleolar proteins and RNAs before immunostaining, which is an established method to detect antigens within the nucleolus (20). Immunostaining with an anti-NP antibody showed that newly synthesized NP was temporarily colocalized with nucleolin/C23, a nucleolar marker, 5 to 7 h postinfection (hpi) (Fig. 1A). The localization pattern of the NP at each time point was as follows: the NP was detected only in the nucleoplasm at 3 hpi, in both the nucleoplasm and the nucleolus at 5 and 7 hpi, and mainly in the cytoplasm at 9 hpi. The localization pattern suggests that nucleolar localization of NP is specific in the early stage of infection and that the NP passes through the nucleolus before nuclear export. Importantly, the NPs were also observed in the nucleolus of cells infected with different virus strains (A/California/04/2009 [H1N1], A/Victoria/361/2011 [H3N2], and A/Udorn/307/1972 [H3N2]) (see Fig. S1 in the supplemental material), indicating that the nucleolar localization of NP is a common phenomenon during the influenza virus life cycle.

10.1128/mbio.03315-21.1 FIG S1 Nucleolar localization of NPs in different strain-infected cells. Subcellular localization of NPs in mock-infected or influenza virus-infected (MOI = 5) cells. Viral strains are labeled at the top of the images. NP and nucleolin were immunostained after protease treatment of fixed and permeabilized cells at 4.5 to 6 hpi. Nuclei are marked by dashed circles. Scale bars, 20 μm. Representative images from duplicate wells are shown. Download FIG S1, PDF file, 0.7 MB. Copyright © 2022 Miyamoto et al. 2022 Miyamoto et al. https://creativecommons.org/licenses/by/4.0/ This content is distributed under the terms of the Creative Commons Attribution 4.0 International license.

To further confirm the nucleolar localization of NP biochemically, we separated virus-infected cells into cytoplasmic, nucleoplasmic, and nucleolar fractions at 4 hpi; α-tubulin (cytoplasm marker), histone H3 (nucleoplasm marker), and nucleophosmin 1/B23 (NPM1, nucleolus marker) were detected in the expected fractions (Fig. 1B). Consistent with the immunostaining data, the majority of NP was detected in the nuclear fraction at 4 hpi, in which similar amounts of NP were detected in both nucleoplasmic and nucleolar fractions. Likewise, a substantial proportion of PB2, PB1, and PA was detected in the nucleolar fraction, as well as in the nuclear fraction. Collectively, our immunostaining and biochemical data demonstrate nucleolar NP localization in the early stage of infection.

### Importance of nucleolar NP localization for functional RNP formation.

**FIG 2:**
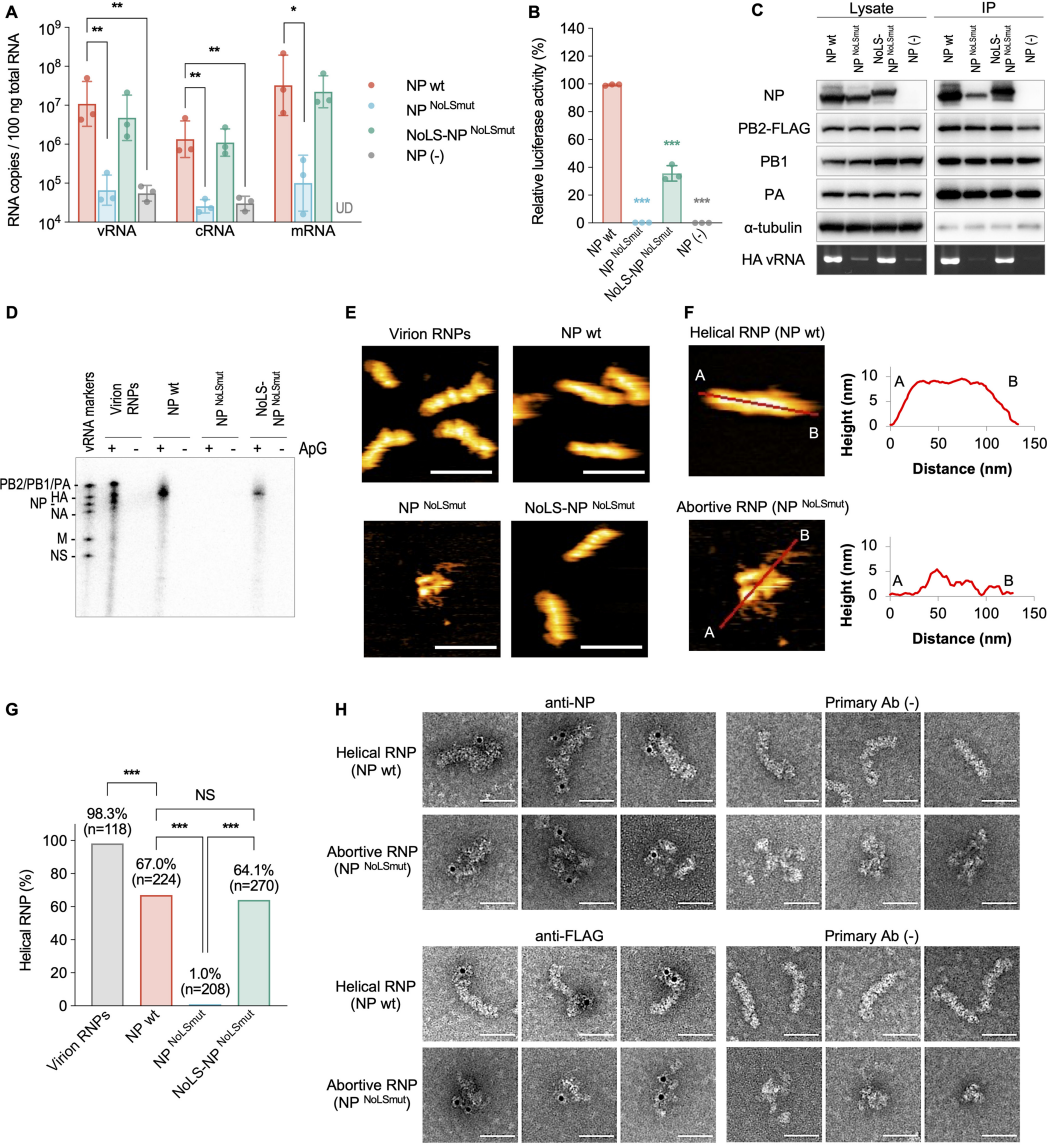
Nucleolar localization of NP is essential for functional and helical RNP formation. (A) Replication and transcription efficiencies of the reconstituted RNPs, measured by strand-specific RT-qPCR. HEK293T cells were transfected with PB2, PB1, PA, an NP proteins and HA vRNA expression plasmids, and total RNA was extracted at 48 h posttransfection (hpt). Their HA vRNA, cRNA, and mRNA copy numbers were compared using a Kruskal-Wallis test with Dunn’s test (*, *P < *0.05; **, *P < *0.01; UD, undetected). The data are presented as geometric means ± the standard deviations (SD) of three independent experiments with two RT-qPCR assays. (B) Polymerase activity of the reconstituted RNPs in HEK293 cells, measured by minigenome assay. Relative firefly luciferase activities were compared to that of the RNPs reconstituted with NP wt using one-way ANOVA with Dunnett’s test (***, *P < *0.001). The data are means ± the SD from three independent experiments with duplicate wells. (C) Reconstruction and immunoprecipitation of RNPs. The RNPs were reconstructed by transient expression of PB2-FLAG, PB1, PA, and NP proteins and HA vRNA in HEK293T cells, followed by immunoprecipitation using anti-FLAG antibody-conjugated agarose beads. The viral proteins and α-tubulin were immunoblotted. The full-length HA vRNA was detected by RT-PCR. Representative images from three independent experiments are shown. (D) *In vitro* transcription of the reconstructed RNPs. Nascent viral RNA was transcribed *in vitro* with ApG primer and detected by autoradiography. RNPs derived from virion (virion RNPs) were used as the control. vRNA markers are the size markers, synthesized *in vitro* by T7 RNA polymerase. A representative image from three independent experiments is shown. (E) HS-AFM observation of RNPs. Representative images of the reconstructed RNPs and the virion RNPs from two independent experiments are shown. Scale bars, 100 nm. (F) Section analysis of the helical and abortive RNPs. (Left) Enlarged HS-AFM images of Fig. 2E. (Right) The heights of the helical and the abortive RNPs were measured at the red lines from A to B. (G) Quantification of helical RNP. The bars show the ratio of helical RNPs in all observed RNPs in HS-AFM analysis. The ratio was compared using one-way ANOVA with Tukey’s test (***, *P < *0.001, NS, not significant). (H) Negative-staining immuno-electron microscopy of the purified RNPs. We analyzed each of the 100 labeled RNPs. The helical RNPs labeled with anti-NP and anti-FLAG antibodies had one to three gold particles, mainly at the terminal region and distributed throughout the RNPs, respectively. The abortive RNPs labeled with anti-NP and anti-FLAG antibodies had one to three gold particles. Of 300 or more RNPs in the primary Ab (–) controls, only one or zero gold particle-bound RNPs were observed. Three representative images are shown. Scale bar, 50 nm.

Of the RNP components, only NP possesses a nucleolar localization signal (NoLS) in addition to a nuclear localization signal (18, 21, 22). To investigate the importance of nucleolar NP localization for RNP formation, we constructed two NoLS-mutant NPs: an NP^NoLSmut^ with alanine substitutions in the NoLS that localizes only in the nucleoplasm and a reverse mutant NoLS-NP^NoLSmut^ with an intact NoLS fused to the amino terminus of NP^NoLSmut^ that facilitates its nucleolar localization (see Fig. S2A and B) (18). Strand-specific RT-qPCR after *in vivo* reconstruction of RNP containing a full-length vRNA demonstrated that the RNPs comprising NP^NoLSmut^ exhibited a significant reduction in vRNA, cRNA, and mRNA production, whereas the RNPs comprising NoLS-NP^NoLSmut^ showed relatively efficient production (Fig. 2A). The results are consistent with those obtained using a luciferase-based minigenome assay (Fig. 2B), as reported previously (18), and further demonstrate that the nucleolar localization of NP is critical for both transcription and replication.

10.1128/mbio.03315-21.2 FIG S2 Nucleolar localization of NPs is critical for transcription and replication. (A) Schematic diagram of NP wt, NP^NoLSmut^, and NoLS-NP^NoLSmut^. NLS1 (residues 3 to 13) and NoLS (NLS2, residues 198 to 216) are represented by light and dark gray shading, respectively. The NoLS motif was added to the amino terminus of NP^NoLSmut^. The alanine replacements are shown in red. (B) Nucleolar localization of the overexpressed NPs in MDCK cells. NP and NPM1 were immunostained after the protease treatment at 10 hpt. Scale bars, 20 μm. Representative images from two independent experiments are shown. Download FIG S2, PDF file, 0.2 MB. Copyright © 2022 Miyamoto et al. 2022 Miyamoto et al. https://creativecommons.org/licenses/by/4.0/ This content is distributed under the terms of the Creative Commons Attribution 4.0 International license.

Since transcription and replication are performed by RNPs, the impact of nucleolar NP localization on RNP formation was elucidated. We coexpressed PB2-FLAG, PB1, PA, and HA vRNA, together with wild-type NP (NP wt) or NP mutant, to reconstitute RNPs in the cells. The cells were then subjected to immunoprecipitation with anti-FLAG antibody, and the precipitates were assessed by Western blotting and RT-PCR (Fig. 2C). NP wt, PB1, and PA were coprecipitated with PB2, and the full-length HA vRNA was also detected in the precipitate (Fig. 2C). In addition, the immunoprecipitated RNPs produced HA mRNA by *in vitro* transcription (Fig. 2D), indicating the assembly of these viral components into functional RNPs. However, NP^NoLSmut^ was barely coprecipitated with PB2, although PB1 and PA were coprecipitated (Fig. 2C). Furthermore, full-length HA vRNA was barely detected in the precipitate, and the immunoprecipitated RNPs did not produce HA mRNA (Fig. 2D), indicating that NP^NoLSmut^ was not properly assembled into functional RNPs, although the heterotrimeric viral polymerase subunit was assembled. Intriguingly, NoLS-NP^NoLSmut^, PB1, and PA were adequately coprecipitated with PB2, from which full-length HA vRNA was detected (Fig. 2C). Moreover, the immunoprecipitated RNPs produced HA mRNA (Fig. 2D), suggesting that some NoLS-NP^NoLSmut^ was assembled into functional RNPs. Taken together, these results indicate that nucleolar localization of NP is indispensable for functional RNP formation.

Ultrastructural analysis of the reconstituted RNPs provided further evidence for the necessity of nucleolar NP localization for assembly into RNPs. Using high-speed atomic force microscopy (HS-AFM), which enables near-native topological ultrastructure visualization of biological specimens, such as influenza virus HA and NP, in solution without any fixation, hydration, and staining (23–26), we investigated the morphology of respective reconstituted RNPs after immunoprecipitation and purification (see Fig. S3A). Approximately 70% of the NP wt-constituted RNPs showed double-helical structure with a uniform height of ∼9 nm (Fig. 2E to G). These RNPs were morphologically indistinguishable from those purified from influenza virions (Fig. 2E; see also Fig. S3B). In contrast, NP^NoLSmut^ was barely assembled into double-helical structures and the resultant RNPs showed pleomorphic morphology with a height of ≤5 nm, where string-like structures, probably naked RNAs based on their structure, were exposed (Fig. 2E to G). Importantly, NoLS-NP^NoLSmut^ was also assembled into double-helical RNPs (∼65% of the RNPs) (Fig. 2E to G). Immuno-electron microscopy confirmed that both double-helical RNPs and the pleomorphic aggregates comprised NP and viral polymerase (Fig. 2H), indicating that the pleomorphic aggregates are abortive RNPs. Taken together, these data demonstrate that nucleolar NP localization is critical for functional double-helical RNP formation.

10.1128/mbio.03315-21.3 FIG S3 Visualization of the reconstructed RNPs. (A) Purification of the reconstructed RNPs using NP wt, NP^NoLSmut^, or NoLS-NP^NoLSmut^. The immunoprecipitated RNPs were further purified by ultracentrifugation through 30 to 70% glycerol gradients. Each fraction was gel electrophoresed and immunoblotted with anti-NP antibody. (B) Supplementary images of the purified RNPs visualized by HS-AFM. Scale bar, 100 nm. (C) Purification of RNPs from the influenza virus-infected cells. The immunoprecipitated RNPs from the PB2-FLAG virus-infected A549 cells were further purified by ultracentrifugation through 30 to 70% glycerol gradients. Each fraction was gel electrophoresed and immunoblotted with anti-NP antibody. (D) Supplementary images of the purified RNPs from the PB2-FLAG virus-infected cells visualized by HS-AFM. Scale bar, 100 nm. Download FIG S3, PDF file, 0.3 MB. Copyright © 2022 Miyamoto et al. 2022 Miyamoto et al. https://creativecommons.org/licenses/by/4.0/ This content is distributed under the terms of the Creative Commons Attribution 4.0 International license.

### Impact of nucleolar disruption on functional RNP formation.

**FIG 3:**
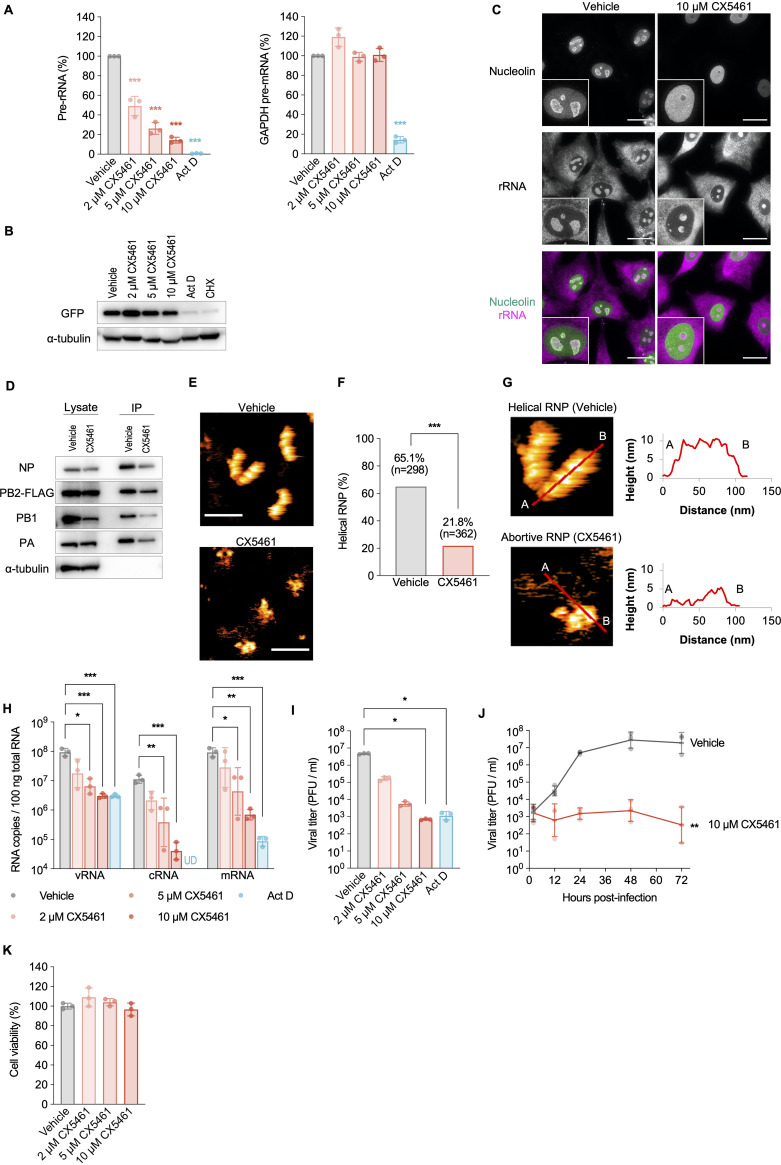
Nucleolar disruption induced by an RNA polymerase I inhibitor impairs helical RNP formation. (A) Selectivity of the RNA polymerase inhibitors on Pol I and II activities. A549 cells were pretreated with CX5461, 10 μg/mL actinomycin D (Act D), or 1% DMSO (vehicle) for 2 h, followed by wild-type virus infection (MOI = 5) for 5 h. Total RNA was extracted and analyzed by RT-qPCR. The expression levels were compared to those of vehicle-treated cells using one-way ANOVA with Dunnett’s test (***, *P < *0.001). The data are presented as means ± the SD of three independent experiments. (B) Effect of CX5461 on mRNA transcription and translation. A549 cells were transfected with a GFP expression plasmid (pCAGGS/GFP) and incubated in medium containing CX5461, 10 μg/mL Act D, 10 μM cycloheximide (CHX), and the vehicle at 12 hpt. After an additional 12-h incubation (24 hpt), the cell lysate was analyzed by Western blotting. Representative images from two independent experiments are shown. (C) CX5461-induced nucleolar disruption. A549 cells were treated with 10 μM CX5461 or vehicle for 8 h. Scale bars, 20 μm. Representative images from three independent experiments are shown. (D) Immunoprecipitation of RNPs from the PB2-FLAG virus-infected A549 cells (MOI = 5), followed by 10 μM CX5461 or vehicle treatment at 2 hpi. The cells were lysed at 4.5 hpi and immunoprecipitated. Representative images from three independent experiments are shown. (E) Representative images of the RNPs in HS-AFM analysis from two independent experiments. Scale bars, 100 nm. (F) Quantification of helical RNP. The bars show the ratio of helical to total RNPs in HS-AFM analysis. The ratio was compared using a Welch *t* test (***, *P < *0.001). (G) Section analysis of the helical and abortive RNPs. Enlarged HS-AFM images of Fig. 3E are shown. Heights of the helical and the abortive RNPs were measured at the red lines from A to B. (H) Effects on viral replication and transcription. A549 cells were pretreated with CX5461, 10 μg/mL Act D, or 1% DMSO (vehicle) for 2 h, followed by wild-type virus infection (MOI = 5) for 5 h. HA vRNA, cRNA, and mRNA copy numbers in the total RNA were measured by strand-specific RT-qPCR and compared using the Kruskal-Wallis test with Dunn’s test (*, *P < *0.05; **, *P < *0.01; ***, *P < *0.001; UD, undetected). The data are presented as geometric means ± the SD of three independent experiments with two RT-qPCR assays. (I) Effect on viral growth. A549 cells were pretreated with CX5461 or 10 μg/mL Act D for 2 h, followed by virus infection (MOI = 0.1). The supernatants were obtained at 24 hpi and subjected to plaque assay. The viral titers were compared using the Kruskal-Wallis test with Dunn’s test (*, *P < *0.05). The data are presented as geometric means ± the SD of three independent experiments. (J) Viral growth kinetics in CX5461-treated cells. A549 cells were pretreated with 10 μM CX5461 or vehicle for 2 h, followed by wild-type virus infection (MOI = 0.1). The supernatants were obtained at 2, 12, 24, 48, and 72 hpi and subjected to plaque assay. The viral titers were compared with those of the vehicle-treated cells using two-way ANOVA (**, *P < *0.01). The data are presented as geometric means ± the SD of three independent experiments. (K) Cytotoxicity of CX5461. A549 cells treated with CX5461 or vehicle for 48 h were subjected to a cell viability assay. The cell viabilities were compared using one-way ANOVA (*P = *0.88). The data are presented as means ± the SD of three independent experiments.

Considering the necessity of nucleolar NP localization for proper RNP formation, nucleolar structure disruption would heavily impact RNP formation. To test this hypothesis, we used a selective RNA polymerase I (Pol I) inhibitor, CX5461 (27); inhibition of Pol I activity that transcribes 47S rRNA (pre-rRNA) causes translocation of some nucleolar proteins to the nucleoplasm, resulting in nucleolar disruption (28, 29). Actinomycin D, which inhibits both Pol I and Pol II activities, was used as the control. RT-qPCR and immunoblotting revealed that CX5461 treatment (2 to 10 μM) inhibited only pre-rRNA transcription (Pol I) without the inhibition of pre-mRNA transcription (Pol II) and translation (Fig. 3A and B), whereas actinomycin D treatment (10 μg/mL) suppressed the transcription of both pre-rRNA and pre-mRNA (Fig. 3A), indicating that CX5461 treatment specifically inhibits Pol I activity. We confirmed that 10 μM CX5461 treatment did not show significant cytotoxicity via, for example, topoisomerase II poisoning (30) (Fig. 3K). In addition to an rRNA staining dye, immunostaining with an antibody against nucleolin, a nucleolar marker, showed that nucleolin in CX5461-treated cells was translocated from the nucleolus to the nucleoplasm in a concentration-dependent manner and that the morphology of the pleomorphic nucleoli was altered into small spherules (Fig. 3C), demonstrating that CX5461 caused nucleolar disruption through Pol I activity inhibition.

To determine whether nucleolar disruption affects RNP formation, A549 cells were infected with a recombinant influenza A virus expressing C-terminal FLAG-tagged PB2 (PB2-FLAG virus) and treated with 10 μM CX5461 at 2 hpi. The RNPs were then immunoprecipitated with an anti-FLAG antibody at 4.5 hpi (Fig. 3D; see also Fig. S3C). CX5461 treatment modestly decreased the amount of immunoprecipitated NP, as well as PB1 and PA subunits, in these cells, although viral protein expression levels were comparable, or marginally lower, compared to those in control cells (Fig. 3D), suggesting that nucleolar disruption impacted RNP formation. Importantly, ultrastructural analysis of the immunoprecipitated and purified RNPs using HS-AFM revealed a significant reduction in efficiency of double-helical RNP formation in CX5461-treated cells (Fig. 3E and F). Most of the RNPs immunoprecipitated from CX5461-treated cells were pleomorphic aggregates (Fig. 3G; see also Fig. S3D) that were similar to the abortive RNPs composed of NP^NoLSmut^ (Fig. 2E and F), while most RNPs immunoprecipitated from control cells had double-helical structures (Fig. 3F). Consistent with the ultrastructural analysis, HA vRNA, cRNA, and mRNA production (Fig. 3H) and viral growth (Fig. 3I and J) were decreased in CX5461-treated cells in a concentration-dependent manner, without any cell toxicity (Fig. 3K). Thus, these results demonstrate that the nucleolus is required for proper assembly of NPs into functional double-helical RNPs.

## DISCUSSION

Influenza virus RNP formation coupled with vRNA replication occurs in the nucleus. However, it remains largely uncertain how nuclear domains are involved in the process. In this study, we showed that the nucleolus is the essential site for formation of functional RNPs with a double-helical structure. At an early infection stage, NP was temporarily localized in the nucleolus. Inhibition of nucleolar NP localization and nucleolar structure disruption affected proper assembly of NPs, resulting in abortive RNP formation. In addition, nucleolar disruption significantly reduced virus replication, as well as genome transcription and replication. These results demonstrated that NP migration into the nucleolus is a critical step for functional double-helical RNP formation and that the nucleolus plays an important role in the influenza virus life cycle.

Using two NP mutants, we showed that nucleolar NP localization via NoLS is required for functional RNP formation (Fig. 2). Because both NP mutants contain alanine substitutions in residues 213, 214, and 216, which are located close to the RNA-binding groove formed by R74, R75, R174, R175, and R221 (NP-G1 region) (31–33), such mutations potentially alter the RNA-binding property of NP and subsequent RNP formation (34). However, NoLS-NP^NoLSmut^, which contains alanine substitutions in the intrinsic NoLS but has an ectopic NoLS at the amino terminus, had the ability to form functional double-helical RNPs (see Fig. S2B and Fig. 2E to G), indicating that the introduction of mutations in the NoLS does not affect the *in cellulo* RNA-binding property of NP for RNP formation. Nevertheless, the RNPs comprising NoLS-NP^NoLSmut^ showed significantly lower polymerase activity than those comprising NP wt (Fig. 2B). This reduction in polymerase activity would be due to the N-terminal-fused NoLS because the NoLS-NP wt mutant, which has an additional NoLS at the N terminus of NP wt (see Fig. S4A), showed significantly lower polymerase activity than NP wt in a minigenome assay (see Fig. S4B). Previously, Ozawa et al. reported that NP-NLS2mut, which is the same mutant as the NP^NoLSmut^ used in our study, exhibits almost no transcription and replication activity (18), which is consistent with our result (Fig. 2B) and confirms that the NoLS is indispensable for functional RNP formation. Oddly enough, Ozawa et al. also show in the same study that NP-NLS2mut is able to support infectious virus-like particle (VLP) formation (18), which seems to contradict the importance of NoLS for functional RNP formation. It remains unclear why infectious VLPs are produced in the absence of functional RNPs. However, one possible explanation is that, because NP^NoLSmut^ forms abortive RNPs together with viral polymerase and vRNA (Fig. 2C and H), such abortive RNPs are incorporated into VLPs. In that study, because cells are infected with mixtures of VLPs and wild-type helper virus, vRNA in the abortive RNP might be transcribed and replicated with the aid of the helper virus, resulting in the detection of infectious VLP formation. In the future, it might be interesting to examine whether abortive RNPs are also packaged into virus particles.

10.1128/mbio.03315-21.4 FIG S4 Effect of additional NoLS fused to N terminus of NP on the polymerase activity. (A) Schematic diagram of NoLS-NP wt. The NoLS motif was added to the amino terminus of NP wt. (B) Polymerase activity of the reconstituted RNPs in HEK293 cells, measured by minigenome assay. Relative firefly luciferase activities were compared to those of the RNPs reconstituted with NP wt using one-way ANOVA with Dunnett’s test (***, *P* < 0.001). The data show means ± the SD from three independent experiments with duplicate wells. Download FIG S4, PDF file, 0.07 MB. Copyright © 2022 Miyamoto et al. 2022 Miyamoto et al. https://creativecommons.org/licenses/by/4.0/ This content is distributed under the terms of the Creative Commons Attribution 4.0 International license.

In our HS-AFM observation, almost all the RNPs purified from virions had helical structures (Fig. 2E; see also Fig. S3B), suggesting that RNPs within the virions exist as helices as reported previously (35, 36). In contrast, 65 to 70% of RNPs reconstituted in plasmid-transfected cells showed helical structures, and the remainder had pleomorphic structures similar to RNPs reconstituted using NP^NoLSmut^ (Fig. 2E and G; see also Fig. S3B). The reason for the reduced proportion of helical RNP formation remains unclear. However, because not only completed RNPs but also nascent RNPs would exist in the plasmid-transfected cells, it is possible that the pleomorphic structures represent these nascent RNPs in the course of assembly.

Nucleolar disruption by a specific Pol I inhibitor disrupted RNP component assembly into functional double-helical RNPs (Fig. 3). Since Pol I-mediated pre-rRNA transcription is required for nucleolar structure maintenance (28, 29), it is possible that certain nucleolar proteins, which are required for vRNA replication-coupled RNP formation, were translocated outside the nucleolus by the Pol I inhibitor treatment. Several host nucleolar proteins, including nucleolin and NPM1, reportedly interact with NP, and some nucleolar proteins, such as nucleolin, NPM1, LYAR, and FMRP, facilitate vRNA replication and RNP assembly (14, 16, 37–39). Thus, inhibition of Pol I activity would change their localizations and disrupt their proper interactions with NP in the nucleolus, resulting in abortive RNP formation.

Several studies have implied the involvement of the nucleolus in vRNA replication. Khatchikian et al. reported that 54 host-28S-rRNA-derived nucleotides are inserted into the HA vRNA during viral replication via genetic recombination (40). This recombination is probably caused by a polymerase jumping mechanism (41, 42), wherein the viral polymerase transitions between HA vRNA and an adjacent host 28S rRNA during vRNA replication. This suggests that the replication occurs at the site of rRNA transcription or at its adjacent site, for example, in or near the nucleolus. An *in situ* hybridization study on salmon anemia virus-infected cells (also belonging to the *Orthomyxoviridae* family) showed nucleolar localization of antigenomic as well as genomic RNA (43). Although the identity of the anti-genomic RNA in the nucleolus remains uncertain, considering that viral mRNA is transcribed in the vicinity of Pol II in the nucleoplasm, the antigenomic RNA likely represents cRNA replicated from vRNA template. In addition to these reports, because the nucleolus exists as a liquid condensate (44) and would result in an increase in the local concentration of NPs, which facilitates NP assembly into the RNP (5), the nucleolus might act as a site of vRNA replication and RNP formation. Although we showed the presence of all viral polymerase subunits, as well as NP, in the nucleolus of virus-infected cells (Fig. 1B), it remains unclear whether NPs are assembled into double-helical RNPs together with the polymerase complex in the nucleolus. Further investigation is needed to identify the site of RNP formation.

In conclusion, we demonstrated that the formation of functional double-helical RNP relies on nucleolar migration of NPs. Our results highlight the importance of the nucleolus during the influenza virus life cycle. Further studies on intranucleolar host factors responsible for RNP formation are necessary to understand the detailed mechanisms of RNP formation, which would contribute to the development of novel antivirals against influenza viruses.

## MATERIALS AND METHODS

### Cell lines.

MDCK cells, kindly provided by Y. Kawaoka (The University of Tokyo), were grown in minimal essential medium (MEM; Thermo Fisher Scientific, Waltham, MA) containing 5% newborn calf serum (16010-159; Thermo Fisher Scientific). Human embryonic kidney 293 (HEK293, CRL-1573) and 293T (HEK293T, CRL-3216) cells and human lung carcinoma (A549, CCL-185) cells were purchased from the American Type Culture Collection (Manassas, VA) and maintained in Dulbecco modified Eagle medium (Merck, Germany) supplemented with 10% fetal bovine serum (FB-1365; Biosera, France). Cultures were maintained at 37°C in a 5% CO_2_ atmosphere. Viruses were grown in MEM containing 0.3% bovine serum albumin (BSA/MEM).

### Plasmid construction.

pCAGGS/NP^NoLSmut^ and pCAGGS/NoLS-NP^NoLSmut^ were constructed using inverse PCR with sequences similar to those previously reported (pCAGGS/NP-NLS2mut and pCAGGS/NLS2-NP-NLS2mut, respectively) (18). To generate pCAGGS/PB2-FLAG, the PB2 open reading frame (ORF) and FLAG (DYKDDDDK) were linked with a linker (AAA). pPol I/PB2-FLAG was constructed by inserting the PB2-FLAG ORF with a stop codon into a truncated pPol I/PB2 plasmid with the 3′ noncoding region and an additional 143 nucleotides of the 5′ terminal coding and noncoding regions (45).

### Inhibitors and antibodies.

The inhibitors used were: CX5461 (CS-0568; ChemScene, Deerpark, NJ), actinomycin D (A1410; Merck), and cycloheximide (037-20991; Fujifilm, Japan). The primary antibodies used for immunofluorescence, Western blotting, and immuno-electron microscopy were as follows: anti-NP mouse monoclonal (46), anti-NP rabbit polyclonal (GTX125989; GeneTex, Irvine, CA), anti-PB2 goat polyclonal (sc-17603; Santa Cruz Biotechnology, Dallas, TX), anti-PB1 goat polyclonal (sc-17601; Santa Cruz Biotechnology), anti-PA rabbit polyclonal (GTX125932; GeneTex), anti-nucleolin rabbit polyclonal (ab22758; Abcam, UK), anti-nucleophosmin mouse monoclonal (ab10530; Abcam), anti-α-tubulin rabbit polyclonal (PM054; Medical and Biological Laboratories, Aichi, Japan), anti-histone H3 rabbit polyclonal (GTX122148; GeneTex), and anti-FLAG mouse monoclonal (M185-A48; Medical and Biological Laboratories) antibodies. The secondary antibodies used were as follows: Alexa Fluor 488-conjugated anti-mouse (A11001; Thermo Fisher Scientific), anti-rabbit (A11008; Thermo Fisher Scientific), and anti-goat (A11055; Thermo Fisher Scientific) antibodies; Alexa Fluor 555-conjugated anti-mouse (A21422; Thermo Fisher Scientific) and anti-rabbit (A21428; Thermo Fisher Scientific) antibodies; HRP-conjugated anti-mouse (NA931; GE Healthcare, Chicago, IL), anti-rabbit (NA934; GE Healthcare), and anti-goat (ab6741; Abcam) antibodies; and 6-nm gold-conjugated anti-mouse (715-195-150; Jackson ImmunoResearch, West Grove, PA) and anti-rabbit (711-195-152; Jackson ImmunoResearch) antibodies.

### Generation of recombinant viruses by reverse genetics.

Reverse genetics was performed using pPol I plasmids containing cDNA sequences of the A/WSN/1933 (WSN; H1N1) viral genes between the human Pol I promoter and mouse Pol I terminator as described previously (47). Briefly, eight pPol I plasmids and pCAGGS protein-expression plasmids for PB2, PB1, PA, and NP were mixed with TransIT-293 (Mirus Bio, Madison, WI) and added to HEK293T cells. At 48 h posttransfection, the cells were treated with 1 μg/mL TPCK-trypsin (Worthington Biochemical, Lakewood, OH) for 30 min and centrifuged at 1,750 × *g* for 15 min at 4°C, and the supernatant was collected and stored at −80°C. PB2-FLAG virus was generated by replacing pPol I/PB2 wt with pPol I/PB2-FLAG plasmid. For subsequent viral amplification, MDCK cells were infected at an MOI of 10^−5^, followed by incubation for 2 days in BSA/MEM containing 1 μg/mL TPCK-trypsin.

### Viral infection.

The WSN, A/California/04/2009 (H1N1), A/Victoria/361/2011 (H3N2), and A/Udorn/307/1972 (H3N2) strains were used in this study. The WSN strain was used unless otherwise stated. Cells were washed with BSA/MEM, inoculated with virus, and placed on ice for 1 h. After removal of the inoculum and addition of fresh BSA/MEM, the infected cells were incubated at 37°C in a 5% CO_2_ incubator. For viral growth analysis, 0.2 μg/mL TPCK-trypsin and 1% dimethyl sulfoxide (DMSO) were added in BSA/MEM. Viral titers were determined by plaque assays using MDCK cells.

### Immunofluorescence.

Cells were plated in 8-well chamber slides (Matsunami, Osaka, Japan) coated with rat collagen I (Corning, Corning, NY). Infected or transfected cells were fixed in 4% paraformaldehyde (PFA) in phosphate-buffered saline (PBS; Nacalai Tesque, Japan) for 10 min and then permeabilized with 0.5% Triton X-100 in PBS for 5 min. The cells were blocked with Blocking One solution (Nacalai Tesque) for 30 min, followed by incubation with primary antibodies overnight at 4°C and secondary antibodies for 1 h at room temperature. For nuclei and rRNA staining, the cells were treated with Hoechst 33342 (Thermo Fisher Scientific) and Nucleolus Bright Red (Dojindo, Japan), respectively, for 10 min. Section images were recorded using DeltaVision Elite (GE Healthcare) with a 60× oil objective and then deconvolved and projected using the Quick Projection tool by softWoRx (GE Healthcare).

### Protease treatment.

Since the optimal condition for protease treatment depends on protease type, lot, and cell strain (20), we recommend verifying the protease concentration and incubation time until the refracted light on the nucleolar surface is almost unable to be observed by light microscopy. After permeabilization, the cells were washed twice in cold PBS on ice and placed in cold 5 μg/mL TPCK-trypsin in PBS. The slides were incubated on a plate incubator (MyBL-P2; AS ONE, Osaka, Japan) at 37°C for 5 min, followed by incubation with cold 4% PFA in PBS (final concentration 2%) on ice for 30 min to terminate the reaction. Thereafter, the cells were washed in PBS and blocked as described above.

### Western blotting.

Western blotting was performed as previously described (17). Briefly, the cells or samples described below were dissolved with 2× Tris-glycine SDS sample buffer (Thermo Fisher Scientific), boiled for 5 min in the absence of reducing agent, and subjected to SDS-PAGE. The proteins were then electroblotted onto Immobilon-P transfer membranes (Merck). The membranes were blocked with Blocking One for 30 min at room temperature and then incubated with primary antibodies overnight at 4°C. After incubation with HRP-conjugated secondary antibodies for 1 h at room temperature, the blots were developed using Chemi-Lumi One Super (Nacalai Tesque).

### Cell viability.

Cell viability was assessed with a CellTiter-Glo luminescent cell viability assay (Promega) according to the manufacturer’s instructions. Briefly, CellTiter-Glo reagent (equal in volume to the culture medium) was added to A549 cells. The plates were shaken on a plate shaker for 2 min to induce cell lysis, incubated at room temperature for 10 min, and subjected to luminescence measurement.

### Minigenome assay.

A plasmid-based minigenome assay was performed as described previously (18). Briefly, HEK293 cells were cotransfected with pCAGGS/PB2, pCAGGS/PB1, pCAGGS/PA, pCAGGS/NP, and pPol I/NP(0)Fluc(0) expressing a firefly luciferase gene-encoding viral minigenome. pGL4.74[hRluc/TK] (Promega) was also transfected as an internal control. At 24 h posttransfection, the luciferase activity was measured using a dual-luciferase reporter assay system (Promega).

### RNP reconstruction and immunoprecipitation.

HEK293T cells were plated in two 10-cm^2^ dishes and transfected using PEI MAX (Polysciences, Warrington, PA) with RNP expression plasmids (3 μg/mL each of pCAGGS/PB2-FLAG, pCAGGS/PB1, pCAGGS/PA, and pCAGGS/NP; 300 ng/μL pPol I/HA). At 2 days posttransfection, the cells were suspended in cold PBS and pelleted by centrifugation at 780 × *g* for 10 min at 4°C. The pellets were resuspended in 500 μL of lysis buffer (50 mM Tris-HCl [pH 8.0], 150 mM NaCl, 5 mM MgCl_2_, 10% glycerol, 0.05% NP-40, 2 mM dithiothreitol [DTT], 10 mM Ribonucleoside-Vanadyl complex [New England Biolabs, Beverley, MA], 1× cOmplete EDTA-free protease inhibitor [Roche]), rotated for 15 min at 4°C, and centrifuged at 20,000 × *g* for 15 min at 4°C. The pellets were resuspended in the buffer and incubated with additional 80 μL of anti-FLAG M2 affinity gel (Merck) on a rotator overnight at 4°C. The gels were washed once with lysis buffer and three times with wash buffer (50 mM Tris-HCl [pH 8.0], 200 mM NaCl, 50 mM Na_2_HPO_4_, 2 mM DTT) and then eluted in 150 μL of wash buffer with 500 ng/μL FLAG peptide (Merck) by rotation on a rotator for 30 min at 4°C. The cell lysates and eluates were electrophoresed on an SDS-polyacrylamide gel and immunoblotted.

### RNP purification.

RNP purification was performed as described previously (48). To prepare virion-derived RNPs, MDCK cells were infected with the virus, followed by incubation at 37°C for 2 days. Virions in the supernatants were purified by ultracentrifugation through a 30% (wt/wt) sucrose cushion. The pellets were resuspended in PBS. The purified virions were lysed in a solution containing 50 mM Tris-HCl (pH 8.0), 100 mM KCl, 5 mM MgCl_2_, 1 mM DTT, 2% lysolecithin, 2% Triton X-100, 5% glycerol, and 1 U/μL RNase inhibitor (Promega) for 1 h at 30°C.

The lysed or immunoprecipitated RNPs were ultracentrifuged through a glycerol gradient (30%–70%) containing 50 mM Tris-HCl pH 8.0 and 150 mM NaCl at 245,000 × *g* for 3 h at 4°C. Each fraction was electrophoresed on an SDS-polyacrylamide gel and immunoblotted with an anti-NP antibody (see Fig. S3A and C). NP-enriched fractions 7 and 8 were used for RNP observations.

### *In vitro* transcription of RNPs.

*In vitro* transcription of RNPs was performed as described previously (26). The purified RNP (0.01 mg/mL) was incubated in a buffer (50 mM Tris-HCl buffer [pH 7.9]; 5 mM MgCl_2_; 40 mM KCl; 1 mM DTT; 10 μg/mL actinomycin D; 1 mM [each] ATP, CTP, and GTP; 0.25 μCi/μL [α-^32^P]UTP; 0.05 mM UTP; 1 U/μL RNasin Plus RNase inhibitor; and 1 mM ApG [IBA, Gottingen, Germany]) at 30°C for 15 min. RNA was purified by using an RNeasy minikit, mixed with an equal volume of 2× RNA Loading Dye (New England Biolabs), heated at 90°C for 2 min, and immediately chilled on ice. The sample was electrophoresed on a 4% polyacrylamide gel containing 7 M urea in 0.5× TBE buffer (Nacalai Tesque) at 120 V for 5 h. The gel was dried at 80°C for 2 h, exposed to an imaging plate (BAS-MS 2025; Fujifilm) for 12 to 24 h, and scanned with a Typhoon 3000 Phosphorimager (GE Healthcare). To prepare vRNA markers, all eight vRNA segments of the influenza virus were transcribed using 0.25 μCi/μL [α-^32^P]UTP and a RiboMAX Large Scale RNA Production System-T7, as described above. The transcribed RNAs were purified and mixed before electrophoresis.

### High-speed atomic force microscopy.

HS-AFM analysis of RNPs was performed as described previously (26). The samples were prepared in a microcentrifuge tube, dropped onto freshly cleaved mica without any surface modification, and incubated for 1 to 5 min at room temperature (∼24°C). The samples on the mica surface were then washed with imaging buffer (50 mM Tris-HCl [pH 7.9], 5 mM MgCl_2_, 40 mM KCl, 1 mM DTT) and observed in the imaging buffer at room temperature (∼24°C) using as SS-NEX high-speed atomic force microscope (RIBM, Tsukuba, Japan). Images were taken at a two images/s using cantilevers (BL-AC10DS; Olympus, Japan) with a 0.1-N/m spring constant and a resonance frequency in water of 0.6 MHz. To increase the resolution, the electron-beam deposited tips were fabricated using phenol or ferrocene powder (49). All HS-AFM images were viewed and analyzed using Kodec software (version 4.4.7.39) (50). A low-pass filter and a flattening filter were applied to individual images to remove spike noise and flatten the *xy* plane, respectively. Rod-like and helical structures with a uniform height of 9.0 ± 1.5 nm were defined as helical RNPs. Pleomorphic nucleic acid-protein aggregates, except for nucleic acids (<2.5-nm height string-like structures) or proteins (<25-nm long globular structures), were defined as abortive RNPs.

### Immuno-electron microscopy.

Purified RNPs were adsorbed onto carbon-coated nickel grids and fixed with 2% PFA for 5 min. The grids were washed, treated with Blocking One, and then incubated with an anti-NP or anti-FLAG antibody overnight at 4°C or for 1 h at room temperature, respectively. After washing, the grids were incubated with 6-nm gold-conjugated anti-mouse or anti-rabbit antibodies for 1 h at room temperature. After washing, the samples were fixed with 2% PFA for 10 min and negatively stained with 2% uranyl acetate solution. The images were acquired with an HT7700 transmission electron microscope (Hitachi High-Tech Corporation, Tokyo, Japan).

### RT-PCR.

Total RNA was extracted using an RNeasy minikit with on-column DNase digestion (Qiagen, Hilden, Germany). Samples (10 ng) of the extracted RNA samples were reverse transcribed using a Uni-12 primer (5′-AGCRAAAGCAGG-3′) and Superscript III reverse transcriptase (Thermo Fisher Scientific). Then, 10-fold-diluted cDNAs were PCR amplified using KOD FX (Toyobo, Osaka, Japan) and 0.25 μM HA segment-specific primers according to the manufacturer’s protocol. The cycling conditions were as follows: initial denaturation for 2 min at 94°C, followed by 25 cycles of 98°C for 10 s, 55°C for 30 s, and 68°C for 2 min. The PCR products were electrophoresed on 1.0% agarose gels containing 0.01% (wt/vol) ethidium bromide in 0.5× TBE. The primers used are listed in Table S1 in the supplemental material.

10.1128/mbio.03315-21.5 TABLE S1 Primer sets for RT-PCR and RT-qPCR. Download Table S1, PDF file, 0.03 MB. Copyright © 2022 Miyamoto et al. 2022 Miyamoto et al. https://creativecommons.org/licenses/by/4.0/ This content is distributed under the terms of the Creative Commons Attribution 4.0 International license.

### RT-qPCR.

Two-hundred nanograms of total RNA was reverse-transcribed using Random primer 6 (New England Biolabs) and Superscript III reverse transcriptase. For qPCR, the reactions contained 1 μL of 10-fold-diluted RT product, 7.5 μL of Thunderbird SYBR qPCR mix, and 0.25 μM concentrations of the primers at a final volume of 15 μL. The cycling conditions were as follows: initial denaturation for 2 min at 94°C, followed by 40 cycles of 98°C for 10 s, 55°C for 15 s, and 72°C for 30 s. The relative expression level of each target genes was normalized to that of GAPDH. The primers used are listed in Table S1. A primer set for pre-rRNA, described previously (51), was used.

### Strand-specific RT-qPCR.

Strand-specific RT-qPCR was performed as described previously (52, 53). Briefly, total RNA was extracted from cells using an RNeasy minikit. cDNAs complementary to the three types of HA genome were synthesized with tagged primers at the 5′ end. A 2.5-μL mixture containing 200 ng of total RNA sample and 20 pmol of tagged primers was heated for 10 min at 65°C, chilled immediately on ice for 5 min, and then reheated to 60°C. After 5 min, 7.5 μL of preheated reaction mixture (2 μL of 5× first-strand buffer, 0.5 μL of 0.1 M DTT, 0.5 μL of dNTP mix [10 mM each], 0.5 μL of Superscript III reverse transcriptase [200 U/μL], 0.25 μL of RNasin Plus RNase inhibitor [40 U/μL, Promega], and 3.75 μL of saturated trehalose) was added, followed by incubation at 60°C for 1 h. For the qPCR, each 15-μL reaction contained 1 μL of 50-fold-diluted RT product, 7.5 μL of Thunderbird SYBR qPCR mix, and 0.25 μM concentrations of primers. The cycling conditions were as follows: initial denaturation for 2 min at 95°C, followed by 40 cycles of 95°C for 10 s and 60°C for 45 s. Tenfold serial dilutions (10^9^, 10^8^, 10^7^, 10^6^, 10^5^, and 10^4^ copies/μL) of synthetic vRNA standards were used to generate a standard curve. The primers used are listed in Table S2.

10.1128/mbio.03315-21.6 TABLE S2 Primer sets for strand-specific RT-qPCR and PCR used to produce templates for *in vitro* transcription of RNA standards. Download Table S2, PDF file, 0.04 MB. Copyright © 2022 Miyamoto et al. 2022 Miyamoto et al. https://creativecommons.org/licenses/by/4.0/ This content is distributed under the terms of the Creative Commons Attribution 4.0 International license.

### Subcellular fractionation.

We performed subcellular fractionation as described previously (54) and optimized the buffers, incubation time, and centrifugal force for MDCK cells. Briefly, pelleted MDCK cells (two 15-cm^2^ dishes) were resuspended in ice-cold mild detergent buffer (20 mM Tris [pH 7.5], 10 mM KCl, 3 mM MgCl_2_, 0.1% NP-40, 10% glycerol) and centrifuged at 100 × *g* for 5 min at 4°C. The supernatants were further centrifuged at 1,400 × *g* for 10 min at 4°C and collected as the cytoplasmic fraction. The pellets were then resuspended in 3 mL of 0.25 M sucrose/10 mM MgCl_2_, layered over a 3-mL cushion of 0.35 M sucrose/3 mM MgCl_2_, and centrifuged at 1,400 × *g* for 5 min at 4°C. The resulting cleaner nuclear pellet was resuspended in 0.35 M sucrose/3 mM MgCl_2_ and sonicated six times for 10 s on ice (10-s rest between pulses) to disrupt nuclei and release nucleoli. The sonicate was layered over a 3-mL cushion of 0.88 M sucrose/3 mM MgCl_2_ and centrifuged at 2,800 × *g* for 10 min at 4°C to pellet the nucleoli, and the supernatant was collected as the nucleoplasmic fraction. All solutions used in the fractionation were supplemented with cOmplete EDTA-free protease inhibitor to minimize protein degradation.

The nucleoli were washed by resuspension in 0.5 mL of 0.35 M sucrose/3 mM MgCl_2_, followed by centrifugation at 2,800 × *g* for 5 min at 4°C. The nucleolar pellet was resuspended in 300 μL of middle-salt RIPA buffer (50 mM Tris [pH 7.5], 300 mM NaCl, 1% NP-40, 0.5% deoxycholate, cOmplete EDTA-free protease inhibitor) containing 16 μL of 1-U/μL RQ1 RNase-free DNase and rotated for 30 min at 4°C. The lysate was centrifuged at 20,000 × *g* for 10 min at 4°C, the supernatant was collected as the nucleolar extract, and the NaCl concentration was adjusted to 150 mM by adding 300 μL of “no salt” RIPA buffer (50 mM Tris [pH 7.5], 1% NP-40, 0.5% deoxycholate, EDTA-free complete protease inhibitor).

The cytoplasmic and nucleoplasmic fractions were mixed in 1× RIPA buffer (50 mM Tris [pH 7.5], 150 mM NaCl, 1% NP-40, 0.5% deoxycholate, cOmplete EDTA-free protease inhibitor) and centrifuged at 2,800 × *g* for 10 min at 4°C. Total protein concentrations were measured using a Pierce BCA protein assay kit (Thermo Fisher Scientific) and adjusted to ∼0.5 μg/mL. The samples (∼0.5 μg) were subjected to Western blotting.

### Statistical analysis.

Prism 9 (GraphPad Software) was used to generate the graphs. The sample size varied per experiment and is indicated in each figure legend. We compared group means by a Welch *t* test, one-way analysis of variance (ANOVA) with Dunnett’s or Tukey’s test, two-way ANOVA, or the Kruskal-Wallis test with Dunn’s test and a Benjamini-Hochberg correction using R packages (55). We considered a *P* value of <0.05 to be statistically significant.

### Data availability.

All data are available from the corresponding author upon request.
