# Supplementary material for: Migration of Influenza Virus Nucleoprotein into the Nucleolus Is Essential for Ribonucleoprotein Complex Formation
Source: mBio. 2022 Jan 4;13(1):e03315-21. doi: 10.1128/mbio.03315-21 (PMC8725578; doi:10.1128/mbio.03315-21)
Supplement: FIG S1 [file mbio.03315-21-sf001.pdf]

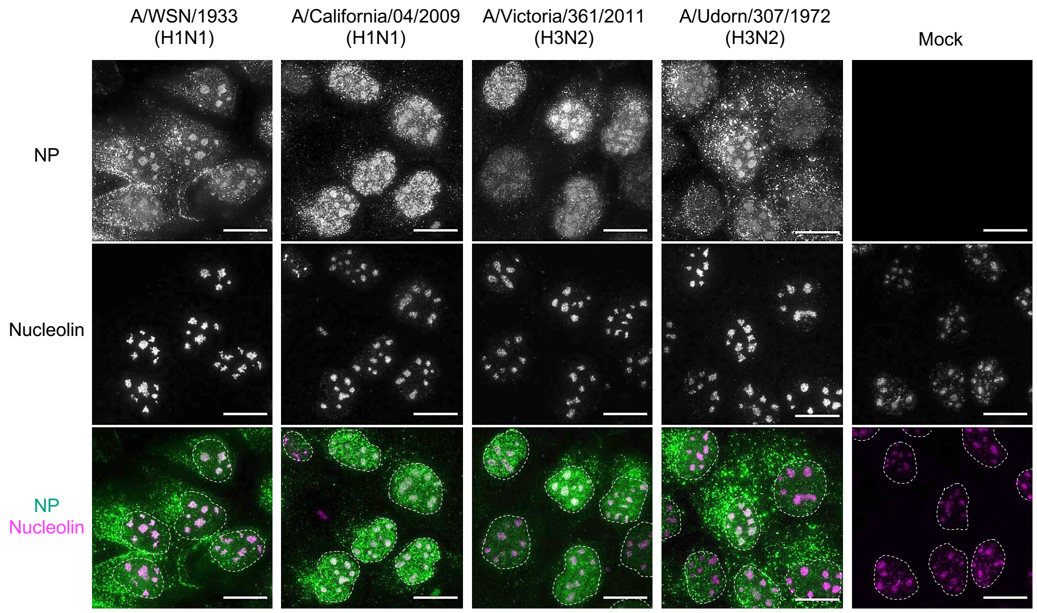

Figure S1. Nucleolar localization of NPs in different strain-infected cells

Subcellular localization of NPs in mock-infected or influenza virus-infected (MOI=5) cells. Viral strains are labelled at the top of the images. NP and nucleolin were immuno-stained after protease treatment of fixed and permeabilized cells at 4.5–6 hpi. Nuclei are marked by dashed circles. Scale bars, 20 μm. Representative images from duplicate wells are shown.
