# Supplementary material for: Migration of Influenza Virus Nucleoprotein into the Nucleolus Is Essential for Ribonucleoprotein Complex Formation
Source: mBio. 2022 Jan 4;13(1):e03315-21. doi: 10.1128/mbio.03315-21 (PMC8725578; doi:10.1128/mbio.03315-21)
Supplement: FIG S2 [file mbio.03315-21-sf002.pdf]

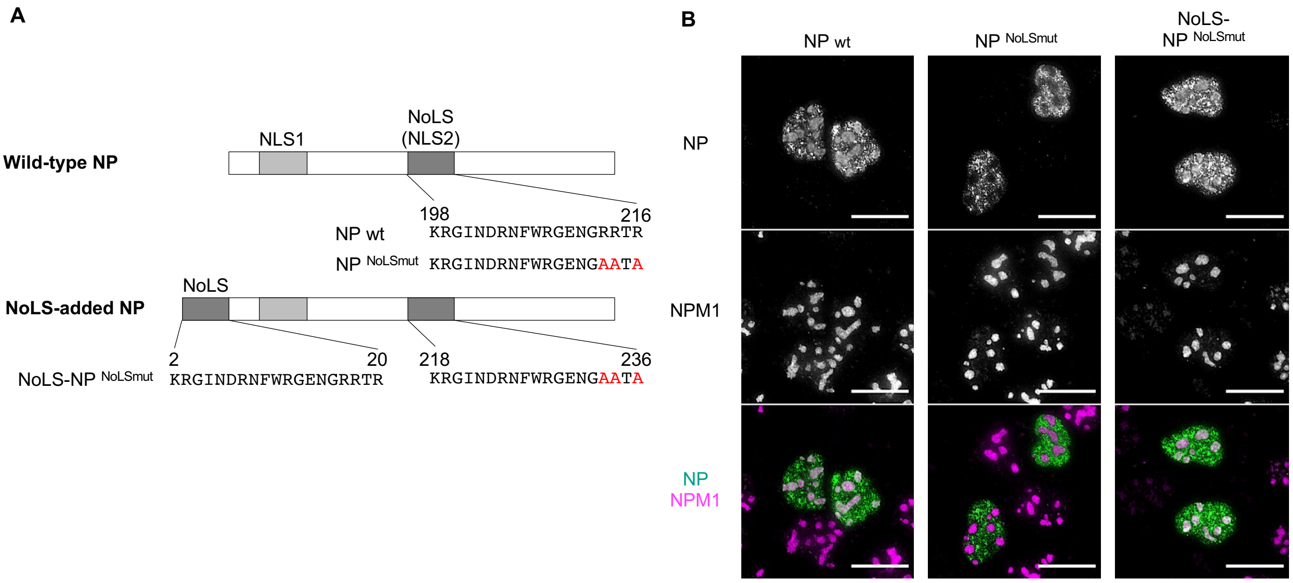

Figure S2. Nucleolar localization of NPs is critical for transcription and replication

(A) Schematic diagram of NP wt, NP^{NoLSmut}, and NoLS-NP^{NoLSmut}. NLS1 (residues 3 to 13) and NoLS (NLS2, residues 198 to 216) are represented in light and dark grey shading, respectively. The NoLS motif was added to the amino terminus of NP^{NoLSmut}. The alanine replacements are shown in red. **(B)** Nucleolar localization of the overexpressed NPs in MDCK cells. NP and NPM1 were immuno-stained after the protease treatment at 10 hpt. Scale bars, 20 μm. Representative images from two independent experiments are shown.
