# Supplementary material for: Migration of Influenza Virus Nucleoprotein into the Nucleolus Is Essential for Ribonucleoprotein Complex Formation
Source: mBio. 2022 Jan 4;13(1):e03315-21. doi: 10.1128/mbio.03315-21 (PMC8725578; doi:10.1128/mbio.03315-21)
Supplement: FIG S3 [file mbio.03315-21-sf003.pdf]

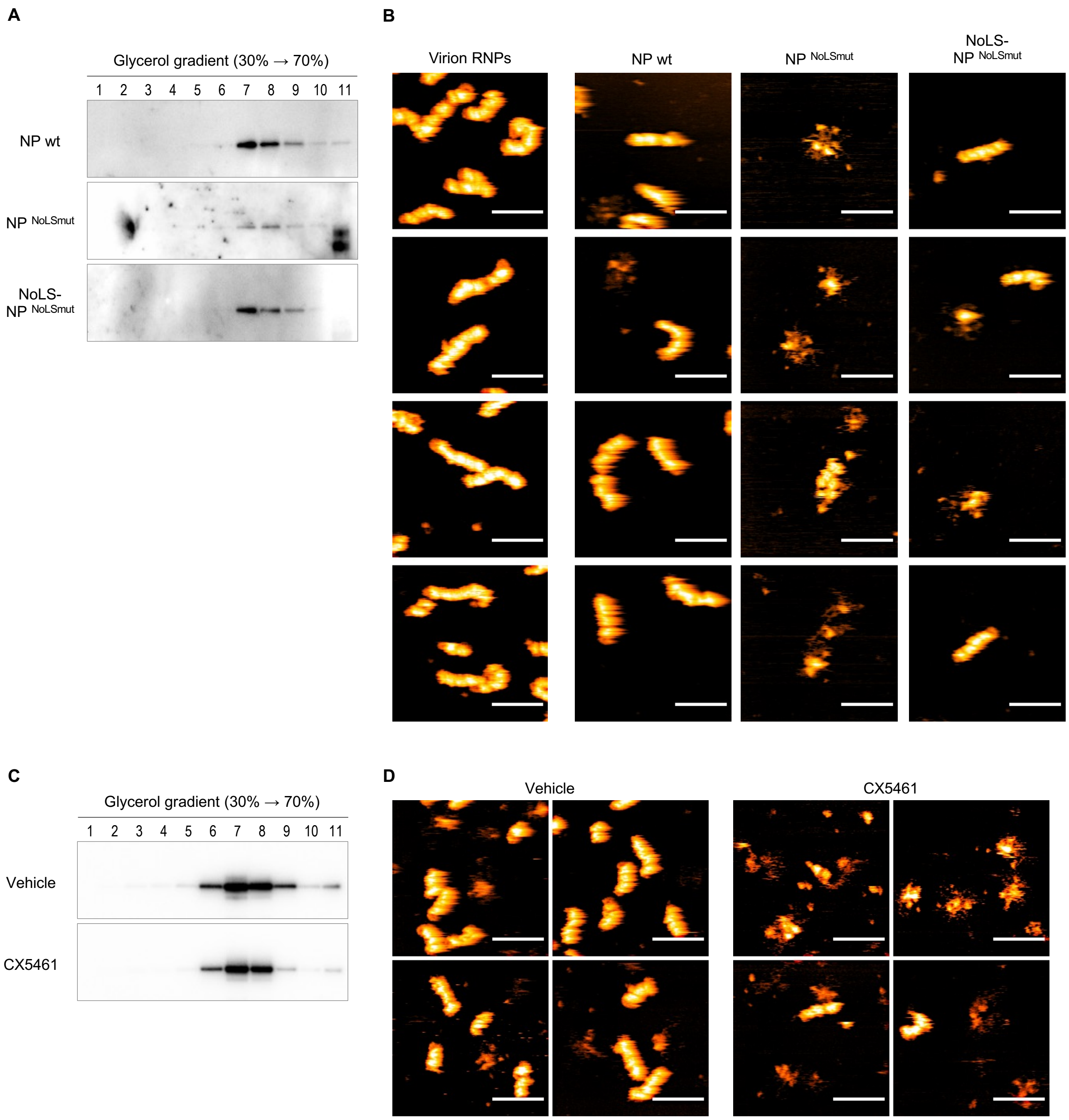

Figure S3. Visualization of the reconstructed RNPs

(A) Purification of the reconstructed RNPs using NP wt, $NP^{NoLSmut}$, or NoLS-$NP^{NoLSmut}$. The immunoprecipitated RNPs were further purified by ultracentrifugation through 30% to 70% glycerol gradients. Each fraction was gel-electrophoresed and immunoblotted with anti-NP antibody. **(B)** Supplementary images of the purified RNPs visualized by HS-AFM. Scale bar, 100 nm. **(C)** Purification of RNPs from the influenza virus-infected cells. The immunoprecipitated RNPs from the PB2-FLAG virus-infected A549 cells were further purified by ultracentrifugation through 30% to 70% glycerol gradients. Each fraction was gel-electrophoresed and immunoblotted with anti-NP antibody. **(D)** Supplementary images of the purified RNPs from the PB2-FLAG virus-infected cells visualized by HS-AFM. Scale bar, 100 nm.
