# Supplementary material for: Migration of Influenza Virus Nucleoprotein into the Nucleolus Is Essential for Ribonucleoprotein Complex Formation
Source: mBio. 2022 Jan 4;13(1):e03315-21. doi: 10.1128/mbio.03315-21 (PMC8725578; doi:10.1128/mbio.03315-21)
Supplement: FIG S4 [file mbio.03315-21-sf004.pdf]

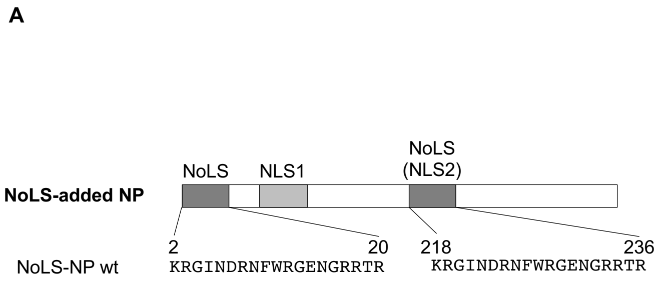

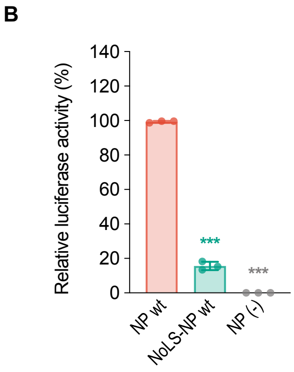

Figure S4. Effect of additional NoLS fused to N-terminus of NP on the polymerase activity

(A) Schematic diagram of NoLS-NP wt. The NoLS motif was added to the amino terminus of NP wt. **(B)** Polymerase activity of the reconstituted RNPs in HEK293 cells, measured by minigenome assay. Relative firefly luciferase activities were compared with that of the RNPs reconstituted with NP wt using one-way ANOVA with Dunnett's test; ***P < 0.001. Data are mean ± S.D., from three independent experiments with duplicate wells.
