# Supplementary material for: Migration of Influenza Virus Nucleoprotein into the Nucleolus Is Essential for Ribonucleoprotein Complex Formation
Source: mBio. 2022 Jan 4;13(1):e03315-21. doi: 10.1128/mbio.03315-21 (PMC8725578; doi:10.1128/mbio.03315-21)
Supplement: TABLE S1 [file mbio.03315-21-st001.pdf]

Table S1.

Primer sets for RT-PCR and RT-qPCR

Name	Sequence (5′ to 3′)
HA_1F	AGCAAAAGCAGGGGAAAATAAAAAC
HA_1775R	AGTAGAAACAAGGGTGTTTTTCCTT
GAPDH_qF	CCATCACTGCCACCCAGAAG
GAPDH_qR	GAAGGCCATGCCAGTGAGCT
Pre-rRNA_qF	CCGCGCTCTACCTTACCTACCT
Pre-rRNA_qR	GCATGGCTTAATCTTTGAGACAAG
GAPDH pre-mRNA_qF	CTTCCAGGAGTGAGTGGAAGAC
GAPDH pre-mRNA_qR	CAGGACCATATTGAGGGACAC
