# Supplementary material for: Migration of Influenza Virus Nucleoprotein into the Nucleolus Is Essential for Ribonucleoprotein Complex Formation
Source: mBio. 2022 Jan 4;13(1):e03315-21. doi: 10.1128/mbio.03315-21 (PMC8725578; doi:10.1128/mbio.03315-21)
Supplement: TABLE S2 [file mbio.03315-21-st002.pdf]

Table S2.

Primer sets for strand-specific RT-qPCR and PCR used to produce templates for *in vitro* transcription of RNA standards

Name	Sequence (5′ to 3′)	Purpose	Target
HA_1F	AGCAAAAGCAGGGGAAAATAAAAAC	RNA standard	HA vRNA
T7_HA vRNA standard_1775R	GGATCCTAATACGACTCACTATAGGGAGTAGAAACAAGGGTGTTTTTCCTTATATTTC		
vRNAtag-HA_881F	GGCCGTCATGGTGGCGAATCTCAAACGCGTCAATGCATGAGTG	RT	
vRNAtag	GGCCGTCATGGTGGCGAAT	qPCR	
HA_993qR	TTGGGCACTCTCCTATTGTGACTG		
T7_HA cmRNA standard_1F	GGATCCTAATACGACTCACTATAGGGAGCAAAAGCAGGGGAAAATAAAAA	RNA standard	HA cRNA
HA_1775R	AGTAGAAACAAGGGTGTTTTTCCTT		
cRNAtag-HA_1775R	GCTAGCTTCAGCTAGGCATCAGTAGAAACAAGGGTGTTTTTCCTT	RT	
cRNAtag	GCTAGCTTCAGCTAGGCATC	qPCR	
HA_1687qF	GGATGTGTTCTAATGGGTCTTTGC		
T7_HA cmRNA standard_1F	GGATCCTAATACGACTCACTATAGGGAGCAAAAGCAGGGGAAAATAAAAA	RNA standard	HA mRNA
HA_dTR	TTTTTTTTTTTTTTTTCCTTATATTTCTGAAATCC		
mRNAtag-HA_dTR	GCCAGATCGTTCGAGTCGTTTTTTTTTTTTTTTTTCCTTATATTT	RT	
mRNAtag	GCCAGATCGTTCGAGTCGT	qPCR	
HA_1687qF	GGATGTGTTCTAATGGGTCTTTGC		
